# Epidemiological science and cancer control

**DOI:** 10.6061/clinics/2018/e627s

**Published:** 2018-09-17

**Authors:** Tatiana N Toporcov, Victor Wünsch Filho

**Affiliations:** Departamento de Epidemiologia, Faculdade de Saude Publica, Universidade de Sao Paulo, Sao Paulo, SP, BR

**Keywords:** Neoplasms, Epidemiology, Early Detection of Cancer, Risk Factors

## Abstract

Epidemiological methods are essential for the discovery of cancer risks and prognostic factors as well as for the evaluation of cancer prevention measures. In this review, we discuss epidemiological surveillance procedures for data collection and processing to guide and evaluate the consequences of anticancer efforts for populations, assess the identification of cancer risk factors, examine barriers to cancer screening and recommended rules for early diagnosis programs. Epidemiological studies have shown that hindrances to cancer information assessment are currently encountered in developing countries. Known cancer risk factors include social determinants, lifestyle factors, occupational exposures, infectious agents, and genetic and epigenetic alterations. Challenges remain in studying the effectiveness of cancer screening; screening can have detrimental effects, and few cancers clearly benefit from screening. Currently, epidemiology faces the challenge of dealing with distinct levels of data, including factors related to social status, lifestyle and genetics, to reconstruct the causal traits of cancer. Additionally, translating epidemiological knowledge into cancer control demands more implementation studies in the population.

## INTRODUCTION

Epidemiology provides information on the distribution of cancer in a population and on cancer determinants and then applies this knowledge to disease control 1. Cancer surveillance, a key attribute of epidemiology and public health practice, provides intelligence data on the burden of different types of cancer in a specified population and, through evidence-based health programs, assesses the success of actions against cancer. Features related to other health outcomes in cancer patients, such as survival after diagnosis and treatment, also fall within the scope of epidemiology and may help define criteria for a strategy against cancer 2. Information regarding diagnosis, treatment and palliative care is widely assessed via clinical epidemiology studies, systematic review methods and meta-analysis models and is required to support evidence-based protocols and design approaches to satisfy clinical priority criteria.

Half of all patients who develop cancer can be cured by currently available treatment resources, but the other half will die from the disease 3. According to the World Health Organization (WHO), approximately 40% of all cancer cases are preventable 4, and considering the global cancer load and the vast resources needed for disease management, public responses have been proposed in many countries. A comprehensive national cancer control plan was designed in the US in the 1990s to minimize the repercussions of cancer on the American population 5. In 2005, the 58^th^ World Health Assembly published a resolution calling on member states to intensify efforts against cancer through control programs 2. Afterwards, the WHO published a guide for cancer control programs 4 that provided practical advice for program managers and policy makers on how to advocate, plan and implement cancer control programs, particularly in low- and middle-income countries. Four basic components were emphasized—prevention, early detection, treatment and palliative care—and evidence-based programs for planning and monitoring cancer control were established.

In this article, we discuss surveillance procedures regarding data collection and processing for evaluating the impact of both cancer and anticancer efforts on populations. We also discuss aspects related to the identification of cancer risk factors, which is a main mission of epidemiology, and examine some cancer screening barriers and recommended rules for early diagnosis programs.

### Assessment of cancer burden

Cancer is a global disease with many disparities among regions, countries and even geographical areas within countries. Several measures are used to estimate cancer burden, including incidence, prevalence, mortality, survival, years of life lost (YLL), years lived with disability (YLD) and disability-adjusted life years (DALY). Cancer surveillance includes systematically measuring cancer parameters, recording and transmitting data, and comparing and interpreting data to detect changes in cancer status in a population 6. Cancer surveillance is based on data from both vital statistics, particularly mortality, and risk factor prevalence surveys.

Cancer registries are important tools for planning and monitoring efforts at all prevention levels 7. Epidemiologists have played a key part in the development and improvement of cancer registries, which began in the US and Germany in the 1920s and currently exist worldwide.

Hospital-based cancer registries record data from cases in specific hospitals and provide information about patients and treatment, aiming to contribute to patient care. These registries do not supply cancer incidence rate statistics but are relevant for appraising other variables, such as cancer patient survival. Population-based cancer registries distinctly emphasize public health demands; these registries collect data on cancer occurrences in delimited populations and furnish valuable information for epidemiological studies and for identifying intervention priorities, especially in resource-limited locations 6.

Reports on global cancer incidence, mortality and prevalence rates are produced periodically by the International Agency for Research on Cancer (IARC). The publication Cancer Incidence in Five Continents (CI5) includes cancer rates from population-based cancer registries worldwide. Almost 100% of the North American population is included in the CI5, whereas less than 10% of the Latin American, Caribbean, Asian and African populations are covered 8. Furthermore, the IARC produces estimates of global cancer statistics through the Globocan project, an interactive web-based platform whose latest version was created in 2012 9.

In the 1990s, the Global Burden of Disease (GBD) Study initiative began, aiming to use the expertise of epidemiologists and other professionals to quantify the comparative magnitudes of health loss over time from diseases, injuries, and risks by age, sex, and population. Currently, the GBD study covers 195 countries and territories and assesses 333 diseases and injuries, 2982 sequelae of these diseases and injuries, and 84 risks or combinations of risks. The GBD study provides information for decision makers at local, regional, national, and global levels 10-12.

Cancer is the second leading cause of death worldwide, causing 8.8 million deaths in 2015. Globally, the incidence of cancer rose to 18.6 million in 2015, and the prevalence, or the number of people living with cancer, was approximately 90.5 million 10,11. More than 60% of cancer cases and approximately 70% of cancer deaths occur in low- and middle-income countries. As a consequence of population aging, the worldwide new cancer cases increased by 33% from 2005 to 2015 13. In this same period, the number of deaths from cancer increased by 17.0%, while the age-standardized death rate decreased by 10% 11. The age-standardized death rate for all cancers decreased in 72% of the countries assessed by the GBD; increasing trends were observed primarily in African countries 13. Between 2005 and 2015, esophageal cancer (-26.8%) and Hodgkin's lymphoma (-23.9%) exhibited the largest reductions in age-standardized death rates, while the death rates due to non-melanoma skin cancer (7.6%) and mesothelioma (7.8%) increased 11.

**Figure 1:**
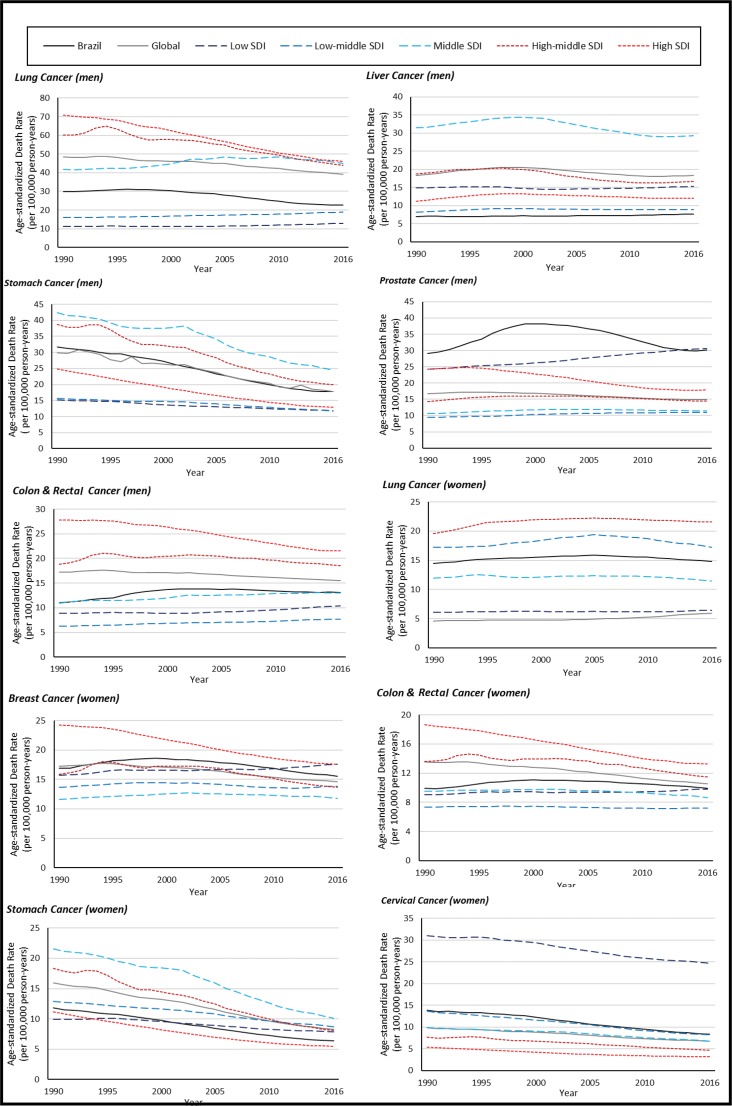
Age-standardized cancer-specific mortality rates in men and women. Source: Global Burden of Disease.

Figure 1 was created using data from the GBD and shows trends in estimated cancer-specific mortalities according to income both worldwide and in Brazil from 1990 to 2016. Disparities are obvious, with pronounced decreasing trends for lung and stomach cancer in men and for colorectal cancer in women in countries with a high or high-middle sociodemographic index (SDI). The SDI is based on the average of the per capita income, average educational attainment, and fertility rates of all areas in the GBD study and is measured on a scale of 0 to 1 11. The values for cervical cancer, which is very preventable, are much higher in low-SDI countries.

Differences in cancer burden according to time or geographical region may generate hypotheses regarding causes of cancer for testing in individual studies. For example, Garland and Garland 14 conducted an ecological study on the correlation between colorectal cancer mortality rates and solar radiation in the US. An inverse correlation, possibly attributable to vitamin D levels, was found. In addition, the potential protective effects of sun exposure and vitamin D levels on other cancer sites was examined 15,16. Further case-control and cohort studies were conducted, and vitamin D and cancer had a consistent inverse causal relationship only for colorectal cancer; however, these results were not confirmed in randomized clinical trials 17. The results of clinical trials assessing the effectiveness of vitamin D supplementation in preventing colorectal adenoma occurrence and recurrence have been inconsistent 18,19. Therefore, knowledge gaps are evident, and more studies testing this hypothesis are necessary.

### Causes of cancer

In a comprehensive study conducted in 1981 encompassing the entire US population in 1978, Doll and Peto 20 attempted to estimate the influence of different cancer causes in a population for the first time. They estimated that approximately 30% of all cancer deaths in the US (43% in men and 15% in women) were due to tobacco smoking. Furthermore, they estimated a similar impact for diet and nutrition and attributed 4% of cancer deaths to carcinogen exposure in the workplace and approximately 10% to infections. The estimates from that study have not changed substantially, even after nearly 40 years 21. Although the percentage of cancer attributable to infectious agents increased to 15.4% worldwide, this increase was not uniform; the percentage was 23.4% in low- and middle-income countries but only 9.2% in more developed countries 22.

Public health measures for controlling both cancer incidence and mortality are feasible since a relatively high proportion of cancer cases may be explained by known risk factors. Approximately 43% of cancer cases in the UK 23 in 2010 were attributable to 12 lifestyle and environmental factors. Estimates for the Brazilian population show that approximately 35% of all cancer cases and between 39% (women) and 46% (men) of deaths will be attributable to known lifestyle and environmental risk factors in 2020 24.

The multifactorial etiology of cancer is an arduous challenge for epidemiologists, considering the complexity of clearly determining human exposure to the factors involved and assessing the interaction of these factors during carcinogenesis. Epidemiological procedures for scrutinizing cancer causes include different types of studies based on sophisticated analysis techniques.

As cancer is a disease with a long period of susceptibility, several strategies have been developed to investigate its causes. The first case-control study on cancer was performed by Janet Elizabeth Lane-Claypon, who collected data via a questionnaire given to 500 hospitalized patients with breast cancer and to an equal number of controls 25. Using simple data presentation and standard statistical procedures, she studied the effect of reproductive experiences in breast cancer etiology 25,26. In 1959, after many case-control studies had been performed on the causes of cancer, Mantel and Haenszel 27 developed statistical methods for risk estimation in retrospective studies; these methods are still in use today.

The first retrospective cohort study was conducted by Bradford Hill in an investigation of workers employed between 1929 and 1938 at a Welsh nickel refinery 28. Hill found that 16 deaths from lung cancer occurred although only one was expected and that 11 deaths from nasal cancer occurred although fewer than one was expected. Currently, evidence from new and more robust studies confirms the role of nickel in lung cancer 29.

The IARC continuously publishes monographs on human carcinogens after reviewing scientific evidence of the causal contributions of chemicals, complex mixtures (e.g., air pollution), occupational exposures, physical and biological agents, and personal habits. For an agent to be considered a human carcinogen, evidence from epidemiological studies and from study models in vivo and in vitro is needed 29,30. The IARC classifies agents under review into four groups according to the evidence for carcinogenicity: carcinogenic to humans (group 1), probably carcinogenic to humans (group 2A), possibly carcinogenic to humans (group 2B), not classifiable as being carcinogenic to humans (group 3), or probably not carcinogenic to humans (group 4) 29. By October 2017, the IARC had classified 120 agents in group 1, 81 in group 2A, and 299 in group 2B. In addition, the IARC publishes the IARC Handbooks of Cancer Prevention, which provide scientific evidence related to reducing the cancer burden 31. Table 1 provides information on factors currently classified as group 1 human carcinogens by the IARC, as well as primary prevention targets to reduce cancer occurrence, as provided in the IARC Handbooks for Cancer Prevention, and the organs affected (adapted from http://monographs.iarc.fr/ENG/Publications/OrganSitePoster.pdf) 32.

#### Social determinants

Global inequalities in cancer burden are patent, with disproportionately high incidences of lifestyle-related cancers in countries classified as having a high human development index (HDI), while infection-related cancers predominate in countries with a lower HDI 33. Individuals in lower educational strata have fewer opportunities to obtain information on cancer self-prevention. Furthermore, most people with cancer in deprived population groups do not have opportunities for cancer diagnosis, and a substantial proportion of cancer cases are diagnosed in people under 50 years of age.

Epidemiological science concerns the way social structures and institutions influence health-related outcomes, including cancer outcomes 34. Such studies have used methodological approaches, such as multilevel analyses, that account for the potential influence of distant disease determinants, such as political and socioeconomic contexts, on proximal factors, e.g., education, diet, tobacco smoking and alcohol drinking. Other strategies, such as mixed methods approaches including both quantitative and qualitative techniques, are also used.

Cancer inequality studies use paradigms based on two distinct values 35. The first paradigm, based on liberty and opportunity, includes studies assessing goods, services and social capital; these studies assess cohesion among individuals via social interactions with family, friends and colleagues via health-related measures. A recent systematic review study, however, has shown that cancer studies present limited evidence of a relationship between social capital and cancer 36. The second paradigm, based on equality and equity, involves social justice studies assessing healthcare and may be used to measure socioeconomic status; these studies assess the impact of lower income or education levels on health-related outcomes. Socioeconomic status is considered a cause of causes since neither cancer incidence nor mortality in low socioeconomic groups are completely explained by known risk factors 37. Positive associations between income and education have been consistently shown at the individual level, and the incidence and mortality of several cancers, such as head and neck 38 and cervical cancer 39, demonstrate that cancer may not be a democratic disease.

The YLL rates for all cancers are lowest in both the lowest and highest sociodemographic groups and are higher for those in the middle socioeconomic stratum. However, people living in countries with the lowest HDI levels die from other conditions before reaching an age at which they can develop cancer.

Lung cancer remains the leading cause of YLL due to cancer in most countries. The YLL rate for lung cancer increased by almost 15% over the past decade, although lung cancer is clearly preventable. For other types of cancer, the geographical pattern is very diverse and possibly reflects differences in risk factors and the capacity of health systems to diagnose and treat the disease 11. Colorectal, breast and pancreatic cancers are mainly responsible for YLL in high-income countries. By contrast, stomach, liver and esophageal cancers are the predominant causes of YLL due to cancer in low-HDI countries 11.

#### Tobacco smoking

A classic example of using epidemiology as a tool to investigate preventable factors associated with cancer is the investigation of tobacco smoke carcinogenicity. The incidence of lung cancer did not become relevant until the 18^th^ century. By the end of the 19^th^ century, mostly in the beginning of the 20^th^ century, deaths due to lung cancer increased in Germany, the UK and the US. In 1898, Herman Rothmann, a medical student, argued that tobacco dust was associated with the increasing incidence of lung cancer. In 1912, in the first monograph on lung cancer, Isaac Adler reported tobacco and alcohol consumption as possible causes of the disease 40. In the 1920s, factors hypothesized to cause the increased lung cancer incidence rates included tobacco smoking and other factors such as pollution, exposure to poisonous gas in World War I and the 1918-1919 global influenza pandemic. Case-control studies performed in Germany, the UK and the US from the 1930s to 1950s 41-44 reported that lung cancer patients smoked more often than did the controls.

In 1952, Doll and Hill assembled a cohort of 40,701 UK doctors and assessed their tobacco smoking history via a questionnaire 45; the participants were followed for 50 years. Since the first results were obtained from this cohort 46,47, tobacco smoking has been consistently recognized as a major cause of lung cancer. In 1986, the IARC recognized tobacco smoke as a carcinogen. According to the IARC 100E monograph, the last full revision of tobacco smoking and cancer, evidence for associations of tobacco smoking with cancer at 20 anatomical sites is available 29,48.

Currently, two-thirds of the world´s population is protected by evidence-based measures proposed by the WHO to contain the tobacco smoking epidemic 49. Brazil is recognized by the WHO for its successful policy against tobacco smoking that has been implemented for the past two decades; other countries worldwide that reported tobacco smoking decreases were also recognized 49. Some studies have shown the proportion of cancer cases and deaths preventable only by eliminating tobacco smoking. Approximately 23% of the cancer deaths and 13% of the cancer cases in Australia in 2013 were attributable to smoking 50, while 19% of the cancer cases in the UK in 2010 23 as well as 30% of the incidence and 35% of the deaths in Japan in 2005 were attributed to smoking 51. In Brazil, 14% of cases in men and 7% of cases in women are predicted to be attributed to smoking by 2020 24. These proportions need to be substantially reduced via tobacco smoking surveillance.

#### Alcohol consumption, diet and physical inactivity

In addition to tobacco smoking, alcohol consumption, unhealthy dietary habits and physical inactivity are the major targets for cancer prevention. Alcohol consumption is included as a dietary habit and was estimated to be responsible for approximately 770,000 cancer cases and 480,000 cancer deaths worldwide in 2012 52. From 2002 to 2012, the proportion of cancers attributable to alcohol intake increased from 3.5% to 5.5%, with higher values for men (7.2%) than for women (3.5%) 52. Studies on alcohol intake as a risk factor for cancer traditionally assessed the type and ethanol content of the alcoholic beverages consumed as well as the duration and frequency of consumption.

Currently, alcohol consumption is recognized to cause cancer of the oral cavity, pharynx, larynx, esophagus, colorectum, liver (hepatocellular carcinoma) and breast (in females) 29. Important differences in cancer risks according to the type of alcoholic beverage consumed are not well supported; all alcoholic beverages contain ethanol, which is considered carcinogenic for humans and is metabolized into acetaldehyde 53, which is also a recognized carcinogen. Studies on gene-environment interactions (GxEs) consistently found a higher risk of alcohol-related cancers among individuals with deficiencies in the oxidation of acetaldehyde to acetate 29. In addition, a recent study on alcohol intake patterns found associations between both binge (heavy episodic) and moderate drinking and breast cancer risk 54. Although moderate alcohol intake might be protective against some other chronic diseases, any alcohol intake might increase the risk of cancer.

Studying the implications of diet on cancer is challenging considering the high possibility of error in measuring exposure. There are substantial differences in the production and preparation of foods in distinct cultures, and dietary patterns are highly heterogenic worldwide. A panel of experts who reviewed the scientific literature on diet and cancer estimated that 30% of all cancer cases are related to diet 55. Observational studies have investigated the influence of food items and groups as well as that of specific nutrients on cancer risks. However, the results from randomized clinical trials did not consistently reproduce the benefits seen in observational studies 56. Studies assessing the role of dietary patterns instead of individual foods have shown that the combined intake of certain foods might be related to cancer risk. However, comparing these results may be difficult when a priori indexing based on dietary recommendations is not performed because of the large variation in dietary patterns across cultures. High consumption of processed meat is recognized as a risk factor for colorectal cancer 57 and has been associated with other cancers, such as breast, esophageal, and gastric cancer and non-Hodgkin's lymphoma 58. Limited evidence for the protective effects of higher fruit intake and nonstarchy vegetables against head and neck cancer 59 and digestive tract cancer is available 60,61. Additionally, the Mediterranean diet has been associated with a lower risk of digestive tract cancers 62. However, more studies assessing dietary and biological markers are needed to elucidate the biological mechanisms involved.

Physical inactivity has been investigated as a cancer risk factor since the 1940s 56. Currently, data on physical activity, which is defined as recreational, commuter, occupational and household activity, are usually collected by self-reports of time spent on a list of activities and are analyzed in separate or joint categories. A recent systematic review of the literature on cancer at 22 body sites, which included 541 studies, found a consistent association between physical activity and decreased incidence and mortality rates of both colon and breast cancer 63.

Recognition of the role of lifestyle factors in cancer may guide interventions to reduce cancer burden. Physical inactivity and unhealthy dietary habits are related to the presence of excess body fat, considered a body mass index (BMI) equal to or greater than 25. In studies using epidemiological designs, interventions to control excess body fat showed sufficient evidence for cancer-preventive outcomes in several cancers 64. Considering the recent worldwide obesity epidemic, preventing cancer and other chronic diseases by controlling body fat is an urgent public health need.

#### Occupational exposures

Occupational cancer epidemiology is notable, as it illustrates not only how epidemiological studies can contribute to the identification of disease risk factors but also how searching for occupational causes of diseases leads to both methodological advances in epidemiological study designs and a better understanding of carcinogenesis. Knowledge about occupational carcinogens is used to establish regulations on exposure limits or agent bans, thus reducing the cancer burden.

While Bernardino Ramazzini had insights into occupational cancer in his De Morbis Artificum Diatriba of 1700, the first consistent evidence of occupational causality in cancer was reported in 1775 by Percival Pott, who noted the occurrence of scrotum cancer among patients who had worked as chimney sweeps at a young age 65. Based on this observation, Pott concluded that their occupation as children or adolescents exposed them to soot, a factor related to the scrotal neoplasia. One hundred and forty years after Pott's original epidemiological description, an experimental model of soot carcinogenesis was reported in 1915 by Yamagiwa and Ichikawa, who described the induction of skin tumors in animals by tar. In the early 1930s, various polycyclic aromatic hydrocarbons were isolated from active coal tar fractions. Finally, in the 1940s, benzopyrene was isolated and identified as the carcinogen responsible for the tumors described by Pott 65-68.

Pott was not only the first to suggest occupational causes of cancer but also the first to introduce the concept of latency, which is defined as the time interval between exposure and disease manifestation or diagnosis and is both a very lengthy period during cancer development and a key concept in the carcinogenesis process. Thus, latency should always be considered in cancer exposure analyses 69.

During the 20th century, numerous carcinogenic agents present in the workplace, including arsenic, asbestos, benzene, cadmium, chromium, nickel, and vinyl chloride, were identified. Noteworthy examples are briefly described.

Problems commonly seen in occupational epidemiology are related to the quality of the exposure assessment. Epidemiologists must pay particular attention to avoiding information bias and confounding, which lead to the underestimation of risks 70. Understanding the causality of diseases is a gradual process that requires specific alternatives using distinct epidemiological study designs. The evolutionary realization of the effects of inhaling asbestos fibers on human health is illustrative of this concept. Although the consequences of inhaling asbestos dust on health have been observed since antiquity, in 1907, Murray was the first to describe asbestosis, the disease responsible for the death of a worker exposed to asbestos fibers during textile spinning 71. In 1935, the pathologist Gloyne noted the carcinogenic potential of asbestos fibers by describing the occurrence of two squamous cell lung carcinoma cases in women with asbestosis 72. This finding was also confirmed by Lynch and Smith 73, who reported a case of lung cancer in a textile worker and included a detailed description of the patient's occupational history. Merewether 74 and Gloyne 72 reported that 13.2 and 14.1% of lung tumors, respectively, were due to asbestosis, as determined by necropsy.

In 1955, the epidemiologist Richard Doll definitively established an association between occupational exposure to asbestos fibers and lung cancer via a retrospective cohort study on mortality 75. Despite the strength of Doll's study, criticism emerged because of the lack of data on smoking; this issue was resolved by Selikoff et al. 76. Simultaneously, evidence of an association between asbestos exposure and pleural mesothelioma emerged in case series studies 77,78. Later, the synergistic effect of smoking and asbestos exposure on lung cancer was identified 79-82.

From the 1950s to the 1970s, asbestos use was widespread worldwide. Since the 1980s, 55 countries worldwide have banned asbestos because of epidemiological and laboratory evidence of its hazards; Brazil was the most recent country to ban the extraction, industrialization and commercialization of asbestos throughout its territory based on a Federal Supreme Court decision made in November, 2017. Unfortunately, the benefits from this ban require more time to be realized, as mesothelioma rates are still high and continue to increase in many European countries given the long latency period between exposure and disease occurrence 11. Increasing trends in mesothelioma mortality have been observed in Brazil, and deaths by mesothelioma are expected to increase and peak in the next decade, approximately one decade after the peak in developed countries 83.

Epidemiological evidence of relationships between organic solvents and cancer has also been important for the control of such occupational carcinogen exposures. An estimated one million people were exposed to solvents in the US in the 1980s, and in the Canadian city of Montreal, 40% of male cancer patients were exposed to solvents 84.

Benzene, commonly used as a solvent, is among the 20 most widely used chemicals worldwide and is identified by the WHO as one of ten chemicals of major public health concern 85. Benzene is a colorless and highly flammable liquid with a sweet smell; this aromatic hydrocarbon is a natural constituent of crude oil and a component of gasoline that has also been identified in industrial emissions and tobacco smoke.

In the past century, the effects of benzene on human carcinogenesis have been evidenced in case-control and cohort studies. Based on results from case-control studies showing a strong relationship between occupational benzene exposure and leukemia, the IARC classified benzene as a human carcinogen in 1982 86. Several subsequent studies have confirmed benzene as a powerful carcinogen. In the last full revision conducted by the IARC, substantial evidence supporting the carcinogenicity of benzene to humans was reiterated, as this chemical was associated with both acute myeloid leukemia and acute nonlymphocytic leukemia. Additionally, positive associations between benzene exposure and acute lymphocytic leukemia, chronic lymphocytic leukemia, multiple myeloma, and non-Hodgkin's lymphoma have been observed 29. Noteworthy results on benzene carcinogenicity emerged from two large cohort studies; the first comprised 1,212 white men employed between 1936 and 1975 to produce Pliofilm, a process requiring large volumes of benzene as a solvent, 87 and the second cohort comprised 74,828 workers recruited from 672 factories in China 88.

Many human carcinogens are occupational carcinogens. Considering evaluations published in IARC monographs, Siemiatycki et al. augmented our knowledge of occupational carcinogens 89 by reporting 28 agents used in several occupations as definite carcinogens, 27 as probable occupational carcinogens, and 113 as possible occupational carcinogens. Therefore, substantial work on occupational carcinogen surveillance and effective interventions must be performed to prevent workplace exposure to these agents.

#### Infectious agents

Studies establishing links between infectious agents and cancer have laid a foundation for prevention. For example, *Helicobacter pylori* (*H. pylori*) is associated with gastric cancer. Based on results from case-control and cohort studies showing that patients with tumors exhibited high proportions of IgG antibodies against *H. pylori*, in 1994, the IARC classified *H. pylori* as a carcinogen related to gastric cancer and gastric lymphoma 90. Rapid advances in molecular biology have allowed microbiome approaches to be used in epidemiological studies, and the association between *H. pylori* infection and gastric cancer has consistently remained. However, recent studies have demonstrated that *H. pylori* infection might be protective against esophageal cancer, increasing the difficulty of preventing cancer by controlling bacteria at the population level 91. Results from meta-analyses have suggested that the eradication of *H. pylori* might prevent gastric cancer and that this approach is most likely more successful in healthy individuals than in those with noncancer gastric diseases or symptoms, although the authors noted that more studies are needed to confirm this conclusion 92,93.

The proportion of infection-related cancer cases is higher in low- and middle- income countries, and prevention by vaccination has been seriously considered. Universal hepatitis B vaccinations began in Taiwan in 1984. Studies including data from Taiwan's National Cancer Registry showed decreased incidences of hepatocellular carcinoma in children after vaccination began 94.

Another successful story in the search for infectious agents related to cancer is the discovery of connections between human papillomavirus (HPV) infection and uterine cervical neoplasms. In the 1980s, zur Hausen isolated and characterized HPV 16 and HPV 18 in uterine cervical biopsies. Since then, studies have been conducted to clarify the causal role of HPV in cervical cancer 95. In a study using PCR to evaluate HPV DNA in 1,000 frozen cervical cancer biopsy samples from America, Africa, Europe and Asia, HPV DNA was detected in 93% of the specimens, and the negative samples showed positive results when reanalyzed with the L1 and L7 primers 96. The association between HPV and cervical cancer is highly dependent on the sensitivity of the laboratory technique used for HPV detection. Thus, advances in molecular biology have increased the validity of the results, which have indeed shown that HPV causes cervical cancer 97.

The association between HPV and other types of cancer, such as anal and oropharyngeal neoplasms, has been investigated, and different causal natures have been shown. Not all oropharyngeal cancers are HPV-related, but HPV infection appears to modify both the disease profile and prognosis 98,99, thus highlighting HPV infection as a disease-modifying entity.

Worldwide, 4.5% of cancers are attributable to HPV infection; the proportion is highest for cervical cancer (83%). The proportion of cancer attributable to HPV infection in women ranges from <3% in Australia, New Zealand and the US to >20% in India and sub-Saharan Africa. In Latin America, this proportion is 13% 100.

In 2012, approximately 15% of all cancer cases worldwide were attributable to infectious agents, and two-thirds of these cancers occurred in less-developed countries. The four infectious agents contributing to most cancers (92%) were *H. pylori*, HPV, hepatitis B virus (HBV) and hepatitis C virus (HCV). Other cancer-related infectious agents include human immunodeficiency virus type 1 (HIV-1), Epstein-Barr virus (EBV), human herpesvirus type 8 (HHV-8), human T cell lymphotropic virus type 1 (HTLV-1), *Opisthorchis viverrini*, *Clonorchis sinensis*, and *Schistosoma haematobium*. Remarkably, in both less- and more-developed countries, infection-attributable cancer incidences are higher in people aged 50 years or younger 22. Undoubtedly, studies on infection and cancer will continue to increase; several other infectious agents have been associated with different types of cancer, but the contributions of these agents to carcinogenesis are not yet completely understood.

#### Molecular biology and genetics

Studies comparing cancer rates in migrants and natives have attempted to elucidate the influences of endogenous risk factors on cancer. Additionally, epidemiological studies assessing cancer in families or comparing cancer risk in twins have attempted to disentangle the association of inherited characteristics with cancer; however, these studies have not completely explained how cancer is attributable to genetics or lifestyle. However, genetic variants are unlikely to account for most cancer cases. Only 5 to 10% of cancer cases are attributable to high-penetrance mutations, such as those in BRCA1 or mismatch repair genes 37.

Currently, advances in genomics and other -omics technologies have allowed the character of genomic and epigenomic variations in humans and pathogens, as well as the relationships of these variations with environmental factors, to be assessed in the setting of carcinogenesis. Epidemiology plays a role in assessing the validity of these technologies when applied to a population 101. Current cancer etiology studies may include measurements of genomic, proteomic, metabolomic, epigenomic, mitochondrial DNA-related, and microbiome-related parameters to better understand disease complexity 101.

GxE assessments are an interesting approach for studying cancer etiology. However, to date, the relationship between GxEs and cancer causality has been poorly established. An example of this relationship, however, is the possible positive association between alcohol intake and genetic polymorphisms in alcohol metabolism-related genes and the risk of head and neck cancers and digestive tract cancers 102-104. Obstacles to the assessment of GxEs are related to the necessity of high-quality longitudinal exposure data and large sample sizes so that a wide range of exposure levels can be studied to obtain statistical power 105.

Another possible avenue for exploring the connections between low-penetrant gene variants and external risk factors is Mendelian randomization, which uses genetic variability as an instrumental variable proxy for the effect of modifiable exposures 106,107. Considering that gene variability distributions are not influenced by environmental exposures in the population, gene variability might be a nonbiased or unconfounded proxy for exposure, thus mimicking a randomized trial and serving as an opportunity to test hypotheses generated in observational studies. Consistent results have been obtained for known risk factors for specific cancers. For example, in studies using this approach, acetaldehyde-metabolizing genes have been linked to head and neck cancer 108, and genetic scores for higher adult BMIs were correlated with increased risks of colorectal, ovarian and lung cancer 109. However, this approach has some limitations, such as the lack of suitable polymorphisms for studying exposures of interest, the failure to establish a reliable genotype-outcome association and the presence of confounding genotype-phenotype disease associations 106.

## EVALUATION OF EARLY CANCER DETECTION METHODS

Screening is a powerful strategy to control some cancers, such as cervical cancer. Herein, we do not aim to review epidemiological evidence or deliver a polemic regarding cancer screening policies; instead, we aim to describe the purpose of epidemiology in investigating the impacts of early diagnosis on cancer control programs.

Disease screening was first conceptualized in 1861 by Horace Dobell, who proposed routine and periodic medical examinations and laboratory tests to discover physiological defects in their earliest state 110. In the 20th century, an emerging belief in the medical community established that a disease course could be altered if an early diagnosis was made 111. In 1907, Charles Childe hypothesized that although cancer is not incurable, delays in diagnosis would make it so, thus proposing that most cancers are curable if diagnosed early. From the 1930s to the 1950s, medical associations began recommending screening based more on intuition than on scientific evidence 111.

In 1968, Wilson and Jugner proposed essential systematic procedural requirements for which diseases and individuals to select for screening programs 112. Forty years later, Andermann et al. 113 published a synthesis of the emerging screening criteria proposed during this period, one of the main principles of which was that the disease needed to be considered an important health problem in the population in order to be eligible for screening.

An adequate study design with proper methods and analysis procedures is key to studying the effectiveness of screening for specific cancers 114, and the challenges of improving the design of screening studies have improved epidemiological methods. Comparing mortality instead of survival between screened and unscreened individuals can prevent lead-time bias, which refers to a finding of increased survival only because of diagnosis anticipation. Another challenge in the design and interpretation of screening studies is that the survival rates of screened patients could be better than the survival rates of patients not screened solely because of their less-aggressive disease, which would explain why cancers are found at an early stage.

To assess the results of screening programs, potential harmful effects should be assessed and balanced against potential benefits. Thus, if early diagnosis does not affect the disease course, early awareness of the disease only makes the individual feel sick for longer. Thus, the harmful impacts of screening should be important considerations for epidemiologists and public health professionals 115.

Cancer screening recommendations have been made by preventive task forces in countries such as Australia, Canada and the US, or by councils such as the Council of the European Union, which suggests evidence supporting current screening for uterine cervical, breast, and colorectal cancers 116-118.

### Current and future challenges in cancer epidemiology

To date, epidemiology has made valuable contributions to cancer prevention and control, some of which have been outlined herein. The risk assessment mission of epidemiology is controversial, but this mission is essential to better understand disease mechanisms; furthermore, it informs control policies. New research pathways, such as the inclusion of big data related to genetics, are required to advance the risk assessment theory in cancer. Genetic epidemiology evaluates the causal mechanisms of cancer while simultaneously considering genes and environmental factors, and Mendelian randomization has emerged as a plausible method for studying the interaction among these factors 106. Consistent analysis of the combined effects of genes and environmental components in cancer studies with large sample sizes is necessary, and establishing international consortia is an approach for overcoming this challenge.

The surveillance of cancer incidence and mortality rates in a population provides awareness for clearly defining policy decision priorities in order to reduce cancer risks. Additionally, these programs must be continuously monitored so that barriers arising from emergent local circumstances can be detected. Thus, many more implementation studies are necessary before these programs can effectively control cancer.

These activities are within the scope of epidemiology, the basic science of public health. However, while such applications are not discussed herein, epidemiology also has broad clinical applications in the study of cancer prognosis. Thus, successful cooperation among epidemiologists, clinicians and laboratory scientists is imperative to better understand cancer etiology, and consequently, cancer control. Primary prevention is the most effective approach for controlling cancer, but preventive initiatives must also be integrated with early diagnosis and proper therapies 37. Furthermore, epidemiologists should collaborate with public health staff and population stakeholders to implement successful cancer prevention and control programs.

## AUTHOR CONTRIBUTIONS

Toporcov TN participated in the conception of the manuscript, conducted the literature review and wrote the manuscript. Wünsch-Filho V conceived the manuscript, wrote and reviewed the manuscript.

## Figures and Tables

**Table 1:** Risk and preventive factors classified as carcinogenic/anticarcinogenic in epidemiological studies and the subsites affected.

Risk factor	Sites affected
**Lifestyle factors**	
Absence of excess body fat	Thyroid, gastric cardia, liver (hepatocellular carcinoma), esophagus, gall bladder, colon and rectum, pancreas, corpus uteri (endometrium), ovary, brain and central nervous system, kidney, multiple myeloma
Aflatoxins	Liver (hepatocellular carcinoma)
Alcoholic beverages	Oral cavity, pharynx, upper aerodigestive tract (acetaldehyde), liver (hepatocellular carcinoma), esophagus, colon and rectum, larynx
Betel quid with tobacco	Oral cavity, pharynx, esophagus
Betel quid without tobacco	Oral cavity, esophagus
Processed meat	Colon and rectum
Smoking cessation (preventive)	Oral cavity, pharynx, stomach, esophagus, pancreas, uterine cervix, larynx, lung, kidney, urinary bladder
Regular physical activity	Colon and rectum
Salted fish, Chinese-style	Nasopharynx
Smokeless tobacco	Oral cavity, esophagus, pancreas
Tobacco smoke, secondhand	Lung
Tobacco smoking	Oral cavity, pharynx, stomach, liver (hepatocellular carcinoma), esophagus, colon and rectum, pancreas, uterine cervix, ovary, nasal cavity and paranasal sinus, larynx, lung, kidney, renal pelvis and ureter, urinary bladder, leukemia/lymphoma
	
**Infectious agents**	
*Clonorchis sinensis*	Biliary tract
Epstein-Barr virus	Nasopharynx, leukemia/lymphoma
Epstein-Barr virus	Leukemia/ lymphoma
*Helicobacter pylori*	Stomach, leukemia/lymphoma
Hepatitis B virus	Liver (hepatocellular carcinoma)
Hepatitis C virus	Liver (hepatocellular carcinoma), leukemia/lymphoma
Human immunodeficiency virus type 1	Anus, uterine cervix, eye, leukemia/lymphoma, endothelium
Human papillomavirus type 16	Oral cavity, tonsils, pharynx, anus, uterine cervix (HPV types 18, 31, 33, 35, 39, 45, 51, 52, 56, 58, and 59), vagina, vulva, penis
Human T cell lymphotropic virus type 1	Leukemia/lymphoma
Kaposi sarcoma herpes virus	Leukemia/lymphoma, endothelium
*Opisthorchis viverrini*	Biliary tract
*Schistosoma haematobium*	Urinary bladder
	
**Medicines**	
Azathioprine	Skin (nonmelanoma)
Busulfan	Leukemia/lymphoma
Cyclophosphamide	Urinary bladder, leukemia/lymphoma
Cyclosporine	Leukemia/lymphoma, skin (nonmelanoma), multiple sites (unspecified)
Diethylstilbestrol (in utero)	Uterine cervix, vagina
Estrogen/Estrogen progesterone menopausal therapy	Corpus uteri (endometrium), ovary
Estrogen-progestogen contraceptives	Liver (hepatocellular carcinoma)
Etoposide with cisplatin and bleomycin	Leukemia/lymphoma
*Phenacetin and analgesic mixtures containing phenacetin*	Renal pelvis and ureter
Tamoxifen	Corpus uteri (endometrium)
Treosulfan	Leukemia/lymphoma
	

Source: International Agency for Research on Cancer. IARC Monographs on Human Carcinogens and Handbooks on Cancer Prevention (https://handbooks.iarc.fr/docs/OrganSitePoster.pdf).
